# TLR2 and TLR9 in Brain Cancer Patients in Relation to EBV Status

**DOI:** 10.3390/ijms27188408

**Published:** 2026-09-21

**Authors:** Michał Brzozowski, Magdalena Góralczyk, Sylwester Bogacki, Małgorzata Polz-Dacewicz

**Affiliations:** 1 Neurosurgery Department, 1st Clinical Military Hospital with Outpatient Clinic in Lublin, 20-049 Lublin, Poland; michal.brzozowski@1wszk.pl; 2 Department of Virology with Viral Diagnostics Laboratory, 20-059 Lublin, Poland; magdalena.goralczyk@umlub.edu.pl; 3 Faculty of Administration and Social Sciences, University of Economics and Innovation, 20-209 Lublin, Poland; sylwester.bogacki@wsei.pl

**Keywords:** TLR 2, TLR 9, EBV, glioblastoma

## Abstract

Toll-like receptors (TLRs) play a significant role in the tumor microenvironment, including in brain tumors, as their activation can both support an anti-tumor response and promote tumor progression through chronic inflammation. However, the precise mechanisms of TLR action have not been fully elucidated, likely due to their complex role in both innate and adaptive immunity. The aim of this study was to analyze serum TLR2 and TLR9 concentrations in patients with glioma in the context of potential Epstein–Barr virus (EBV) infection. TLR9 levels were also analyzed in relation to the presence of viral DNA and EBER molecules in tumor tissue, as well as EBV-related serological responses. The results demonstrate that TLR2 and TLR9 levels were higher in patients with glioblastoma than in control groups. Notably, however, lower TLR9 concentrations were observed in patients testing positive for EBV. Furthermore, a detailed data analysis revealed a link between TLR9 levels and the presence of EBER molecules and/or EBV DNA in tumor tissue. Nevertheless, given the undefined role of EBV in the pathogenesis of glioblastoma, further intensive research in this area is required.

## 1. Introduction

Glioblastoma (GB) is the most aggressive primary malignant tumor of the central nervous system; despite advances in surgery and radiotherapy, it is associated with a poor prognosis and high mortality [1,2]. The overall survival time for newly diagnosed GB undergoing standard treatment is approximately 14–16 months, and the 5-year survival rate is less than 7% [3]. In elderly patients (≥70 years of age), median survival is less than 10 months [4].

The biological behavior of GB is determined not only by the intrinsic properties of tumor cells but also by complex interactions within the tumor microenvironment [5], which play a particularly important role in tumor progression, immune evasion, and the response to treatment. Therefore, identifying factors that modulate innate and adaptive immune responses in GB may provide new insights into the mechanisms underlying tumor development and persistence.

The innate immune system provides the first line of defense against viral infections through the recognition of pathogen-associated molecular patterns [6,7,8].

Toll-like receptors (TLRs), whose family comprises 10 functional members in humans (TLR1–TLR10), are key pattern-recognition receptors (PRRs); they detect conserved microbial structures, i.e., pathogen-associated molecular patterns (PAMPs) and damage-associated molecular patterns (DAMPs), and initiate the innate immune response by triggering intracellular signaling pathways by which the organism produces inflammatory mediators and antiviral molecules [9,10].

Human TLRs can be divided into surface receptors, which primarily recognize microbial components such as lipoproteins, lipids, and proteins (TLR1, TLR2, TLR4, TLR5, TLR6, and TLR10), and endosomal receptors, located primarily in endosomal compartments, which recognize microbial nucleic acids such as viral RNA and DNA (TLR3, TLR7, TLR8, and TLR9) [11,12,13].

Upon recognizing specific ligands, individual TLRs activate a signaling pathway dependent on MyD88 or TRIF, leading to the secretion of various cytokines. Furthermore, TLRs play a key role in the maturation of dendritic cells (DCs), which serve as a link between innate and adaptive immune responses.

The expression of all ten TLRs has been detected in the human brain, and in particular, the increased expression of TLR1, TLR2, TLR4, TLR5, TLR6, and TLR9 has been observed in glioma [14,15,16].

TLR2 is a transmembrane protein encoded by a gene located at locus 4q31.3 on chromosome 4. It is the only Toll-like receptor that forms complexes (heterodimers) with more than two other Toll-like receptors (TLR1, TLR6, TLR4, and TLR10). TLR2 is found primarily on the surface of immune cells—such as monocytes, macrophages, dendritic cells, B and T lymphocytes, granulocytes, and neutrophils—as well as many other cell types, for example, cardiomyocytes, epithelial cells, and endothelial cells [17].

TLR9, encoded by a gene located at the 3p21.3 locus, is an intracellular endosomal receptor and was first cloned in 2000 [18]. It is capable of recognizing unmethylated CpG-type DNA of bacterial, viral, and parasitic origin, as well as self-DNA within immune complexes [19].

A positive correlation has been demonstrated between TLR2 mRNA and protein levels in glioma tissues, and the grade of histological malignancy [20]. Furthermore, the overexpression of this protein may enhance glioma cell activity and cell cycle progression.

TLR9 expression was investigated in human glioma cell lines (U251 and U87), primary human glioma biopsy samples, and isolated human glioma stem cells (GSCs) and was found to be elevated and correlated with a higher grade of glioma malignancy and a poorer prognosis [15,16,21].

Among these receptors, TLR9 plays a particularly important role in the recognition of DNA viruses. It is primarily localized within endosomal compartments and recognizes unmethylated cytosine–phosphate–guanine (CpG) motifs present in microbial and viral DNA [7]. Upon ligand recognition, TLR9 recruits the adaptor protein MyD88 and activates downstream signaling pathways involving IRAK and TRAF6 proteins, ultimately leading to the activation of transcription factors such as nuclear factor κB (NF-κB) and interferon regulatory factor 7 (IRF7). This cascade can stimulate the production of type I interferons and pro-inflammatory cytokines, thereby contributing to antiviral immune responses.

Epstein–Barr virus (EBV), which is widespread in the human population, has been proven to contribute to the development of various cancers [22,23].

The tumor microenvironment (TME) is a complex ecosystem in which immune cells coexist with tumor cells. Growing evidence indicates that EBV can modify the TME to its own advantage, creating immunosuppressive conditions that favor the development and progression of virus-associated tumors [24].

We selected two distinct TLRs for our study, TLR2 and TLR9, and analyzed their serum concentrations in patients with glioblastoma in the context of potential Epstein–Barr virus (EBV) infection. We also analyzed TLR9 levels in relation to the presence of viral DNA and EBER molecules in tumor tissue, as well as the serological response against EBV.

## 2. Results

### 2.1. Subgroups of Patients Who Qualified for This Study

**Figure 1:**
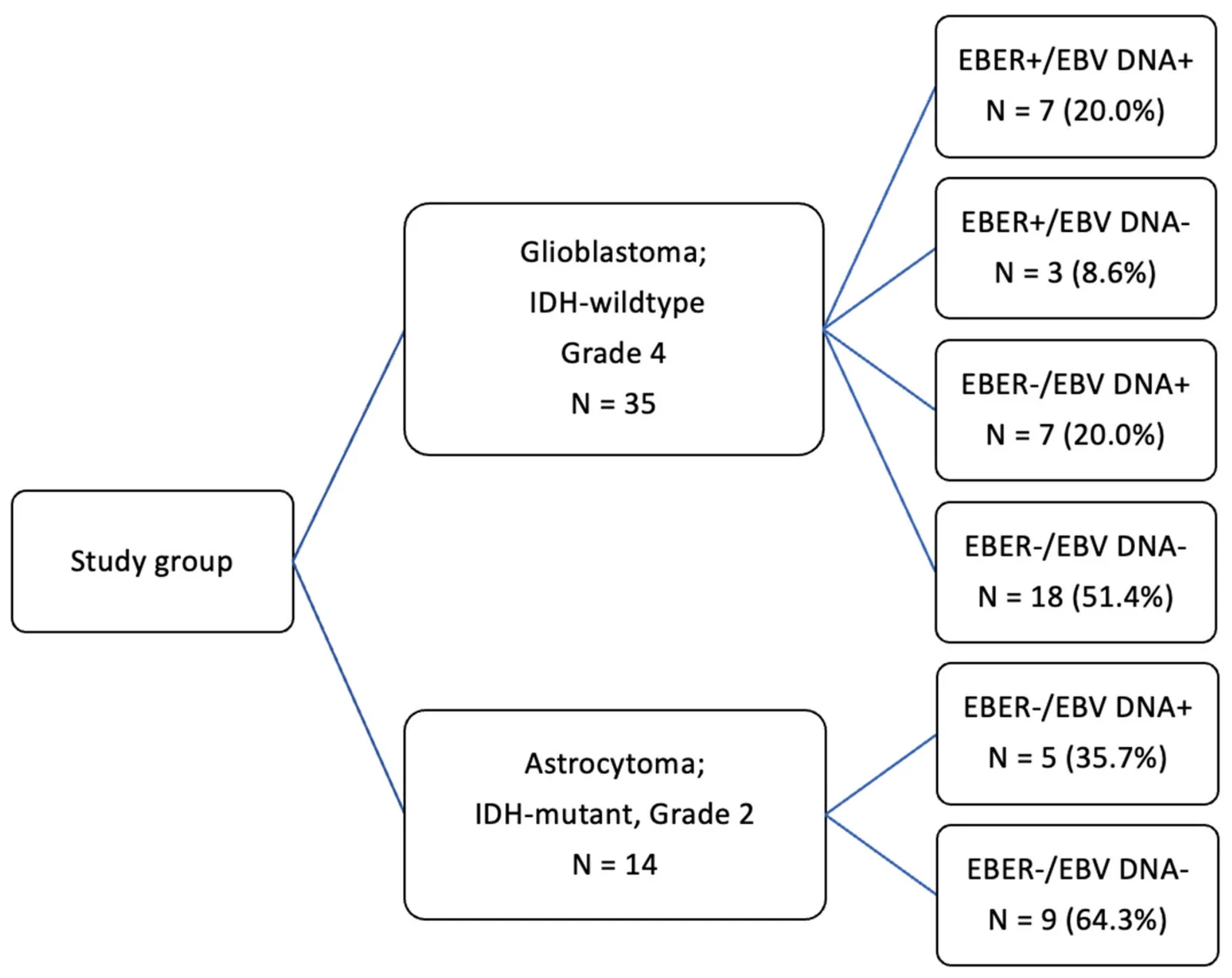
Subgroups of patients who qualified for this study.

The study group consisted of 49 patients with brain cancer, where 35 presented with glioblastoma and 14 with astrocytoma. Following histopathological and EBER assessment performed by an experienced hospital pathologist, we received clinical samples of tumor tissue, where 10 glioblastoma samples were EBER-positive, and 25 were EBER-negative, while all astrocytoma samples were EBER-negative. All received specimens were subsequently analyzed in our laboratory for EBV DNA. Figure 1 illustrates the detailed division of patients into subgroups.

TLR2 and TLR9 concentrations and antibody levels were analyzed in patient serum. Additionally, TLR2 and TLR9 concentrations in tumor tissue were assessed.

### 2.2. Serum TLR2 and TLR9 Concentrations in Brain Cancer Patients Compared with the Control Group

**Figure 2:**
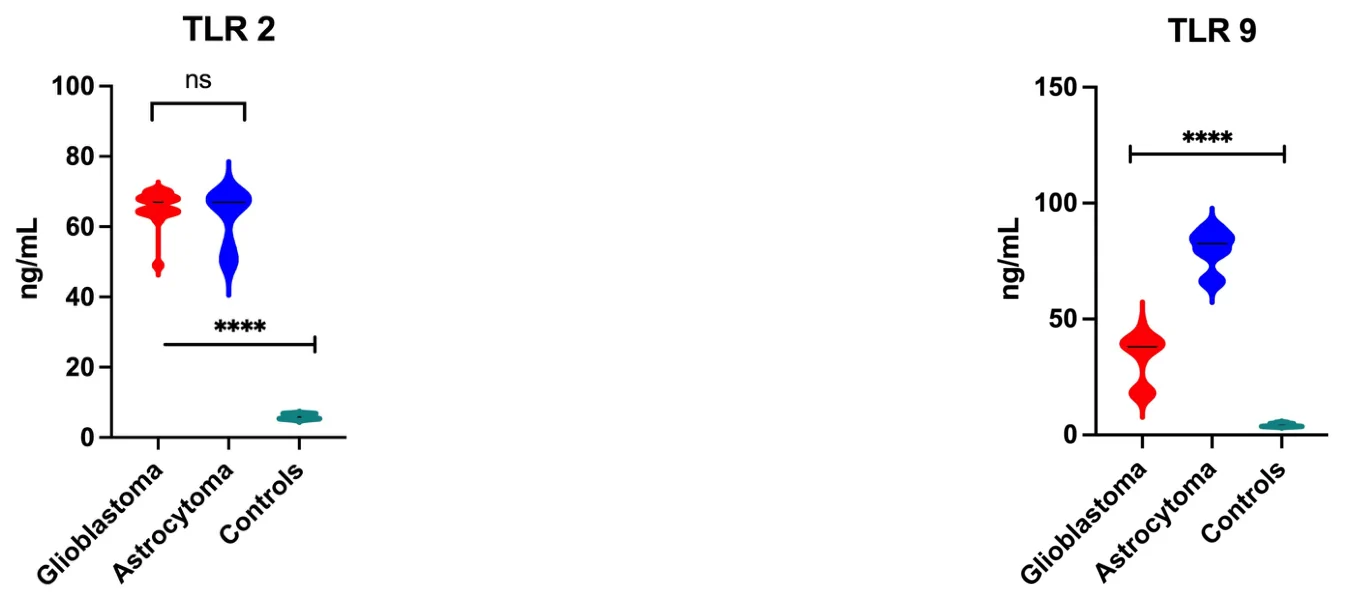
Serum TLR2 and TLR9 concentrations in patients with glioblastoma, with astrocytoma, and in the control group. **** *p* < 0.0001.

In the first stage of our study, we compared serum concentrations of TLR2 and TLR9 in patients diagnosed with glioblastoma and astrocytoma against a control group (Figure 2).

Our study showed that the serum concentrations of TLR2 and TLR9 were higher in patients with brain cancer compared with the control group (*p* = 0.0001). Serum TLR2 concentrations in patients with glioblastoma and astrocytoma did not differ significantly: 65.5 ng/mL and 62.9 ng/mL, respectively. In turn, the serum TLR9 concentrations in glioblastoma patients were lower than in astrocytoma patients: 33.5 ng/mL and 80.1 ng/mL, respectively (*p* = 0.0001). In the control group, the concentrations of both TLRs were low, amounting to 5.9 ng/mL TLR2 and 4.3 ng/mL TLR9. Detailed data are presented in Table S1 in the Supplementary Materials.

### 2.3. Evaluation of Serum TLR2 and TLR9 Concentrations in the Studied Subgroups of Glioblastoma and Astrocytoma Patients

**Figure 3:**
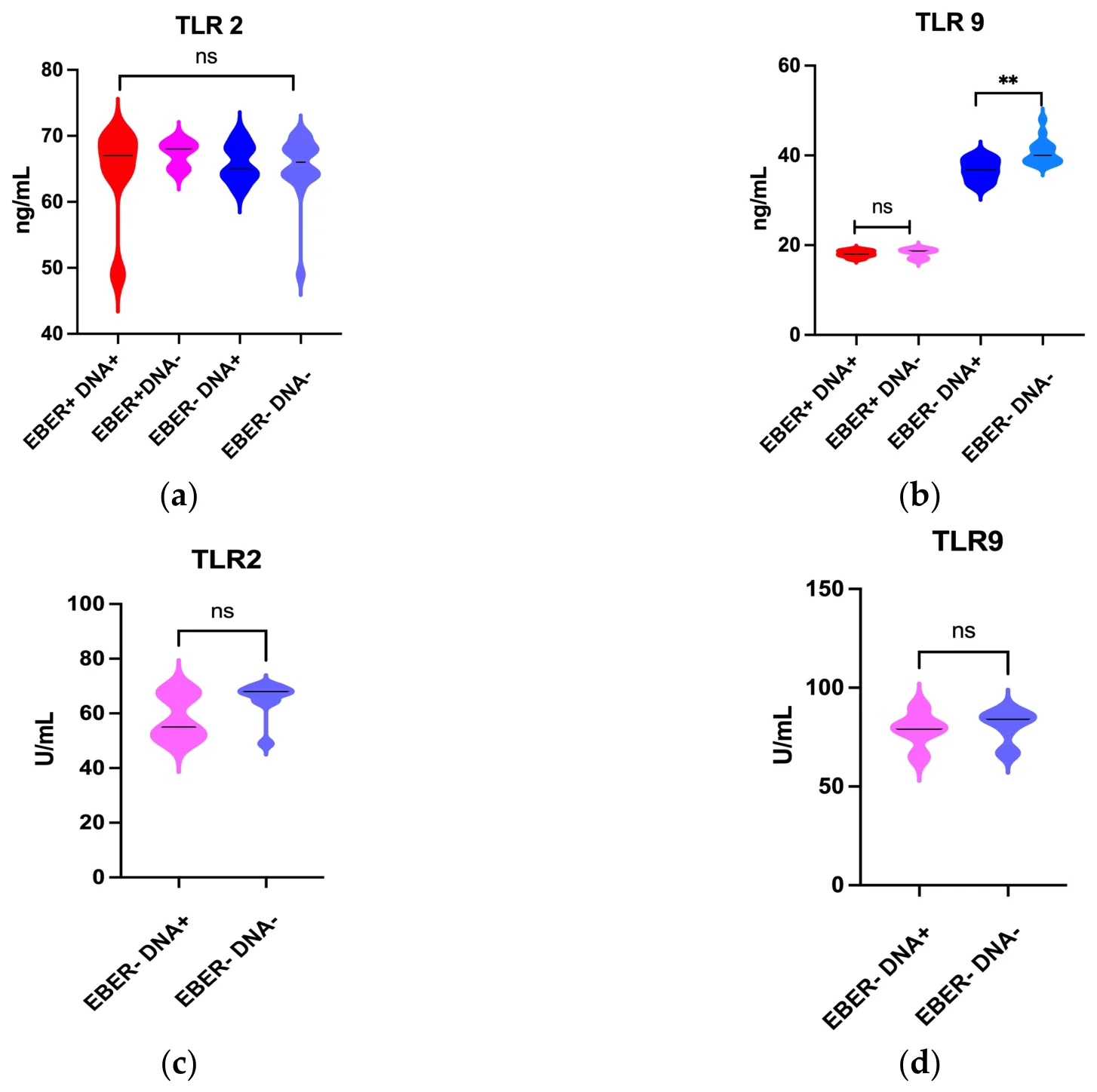
Serum concentrations of TLR2 (**a**) and TLR9 (**b**) in glioblastoma patients and TLR2 (**c**) and TLR9 (**d**) in astrocytoma patients in the presence of EBER and EBV DNA. ** *p* = 0.001.

In the next stage of this study, the aim was to determine the serum concentrations of both TLRs in specific subgroups of patients with glioblastoma (Figure 3a,b) and astrocytoma (Figure 3c,d).

The analysis showed that the presence of EBV did not affect TLR2 levels, which were similar across all analyzed subgroups, with slight differences that were not statistically significant.

On the other hand, TLR9 concentrations differed significantly between the subgroups. The lowest TLR9 concentration was recorded in the serum of patients whose tumor tissue tested positive for EBER, regardless of the presence or absence of EBV DNA. Conversely, lower TLR2 concentrations were observed in EBER-negative samples where EBV DNA was present. Detailed data are presented in Tables S2 and S3 in the Supplementary Materials.

In turn, EBER was not detected in any of the samples from patients with astrocytoma. Therefore, only two subgroups were analyzed, i.e., EBER-DNA+ and EBER-DNA-. The serum concentrations of neither TLR2 nor TLR9 differed significantly between the two analyzed groups. Detailed data are presented in Tables S2a and S3a in the Supplementary Materials.

### 2.4. Evaluation of EBV Load in the Studied Subgroups of Glioblastoma Patients (EBER+ and EBER-)

**Figure 4:**
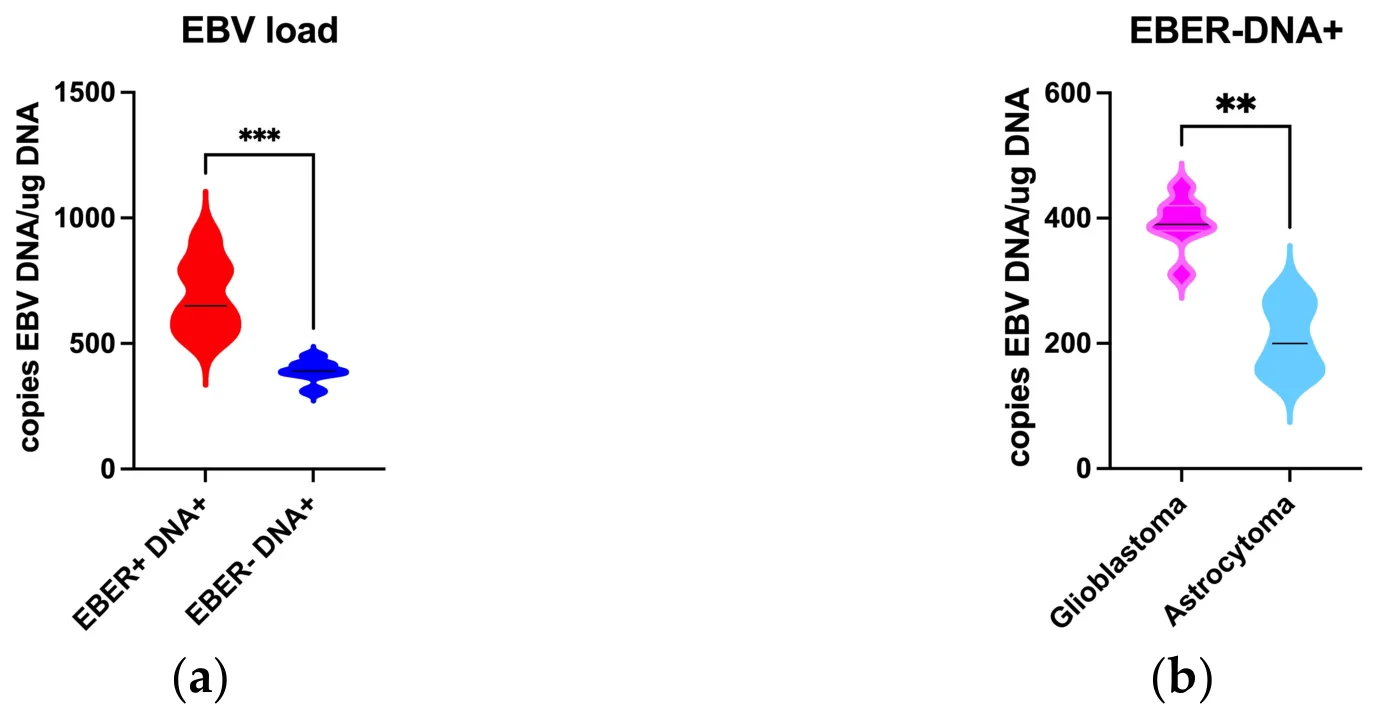
(**a**) EBV DNA load (copies/µg DNA) in glioblastoma tissue in relation to the presence of EBER and DNA. (**b**) Comparison of glioblastoma and astrocytoma EBER-DNA+ samples. ** *p* = 0.001, *** *p* = 0.0001.

The EBV load in tumor tissue was also evaluated in two subgroups of glioblastoma patients, i.e., EBER+ and EBER- (Figure 4a). Significantly higher EBV loads were detected in EBER-positive samples.

All astrocytoma cases were EBER-negative. Therefore, we compared the EBV load in glioblastoma and astrocytoma tissues (EBER-negative/EBV DNA-positive) (Figure 4b). This comparison showed a higher EBV load in glioblastoma than in astrocytoma, despite the absence of EBER in the tumor tissue. Detailed data are presented in Table S4 in the Supplementary Materials.

### 2.5. Assessment of Anti-EBV Antibody Levels in the Studied Subgroups of Glioblastoma Patients

**Figure 5:**
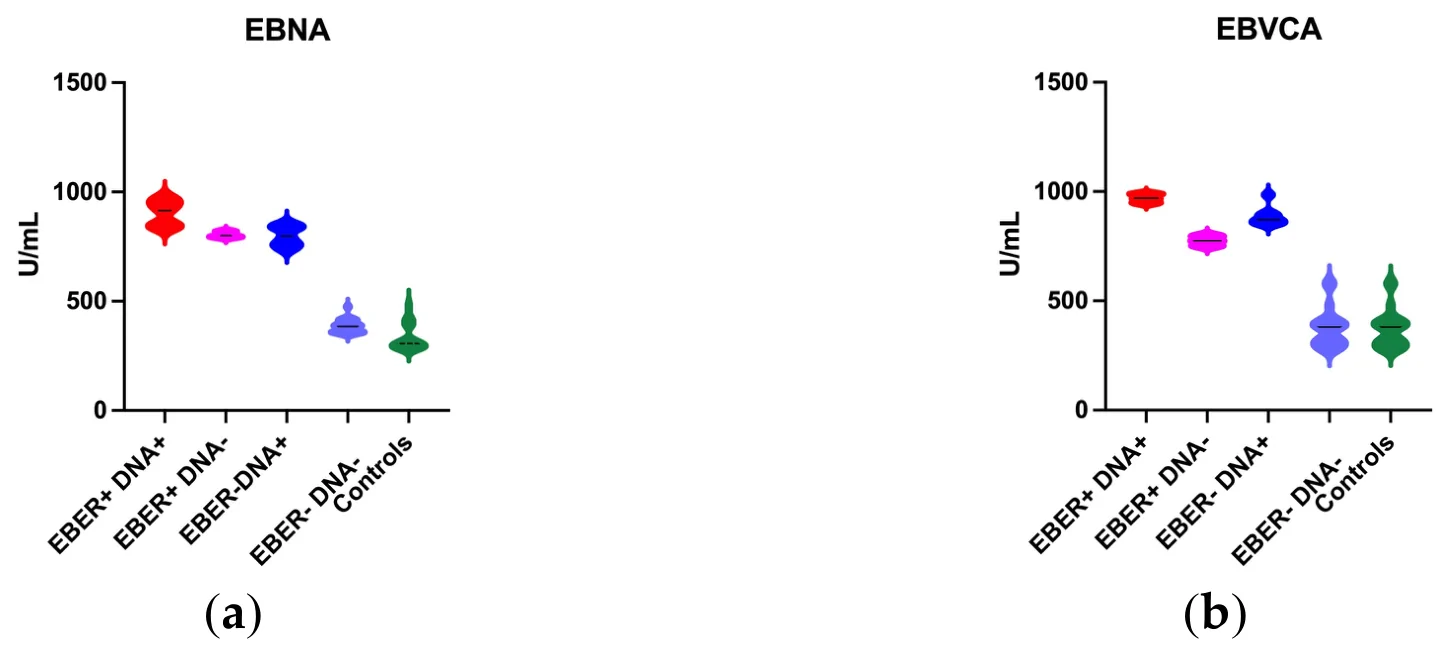
The levels of anti-EBNA IgG (**a**) and anti-EBVCA IgG (**b**) antibodies.

We next analyzed the levels of two main types of IgG antibodies against EBV: anti-VCA and anti-EBNA (Figure 5). The results were compared with a control group.

In the EBER-DNA-negative patient subgroup, EBNA (Figure 5a) and EBVCA (Figure 5b) antibody levels were similar to those in the control group and lower than in the other GB subgroups.

The highest EBNA antibody level (903.1 U/mL) was observed in the EBER+ DNA+ subgroup, followed by the EBER+ DNA− subgroup (803.7 U/mL) and the EBER− DNA+ subgroup (798.2 U/mL). Detailed data are presented in Table S5 in the Supplementary Materials.

For anti-EBVCA antibodies, the values in the individual subgroups were as follows: 969.2 U/mL, 775.1 U/mL, and 888.1 U/mL. A detailed analysis is provided in Table S6 in the Supplementary Materials.

### 2.6. Analysis of Correlation of EBV Load in Tumor Tissue with TLR9 and Anti-EBNA Levels

Given that serum TLR2 levels did not differ among the subgroups of glioblastoma patients, only TLR9 was included in the subsequent analysis.

**Figure 6:**
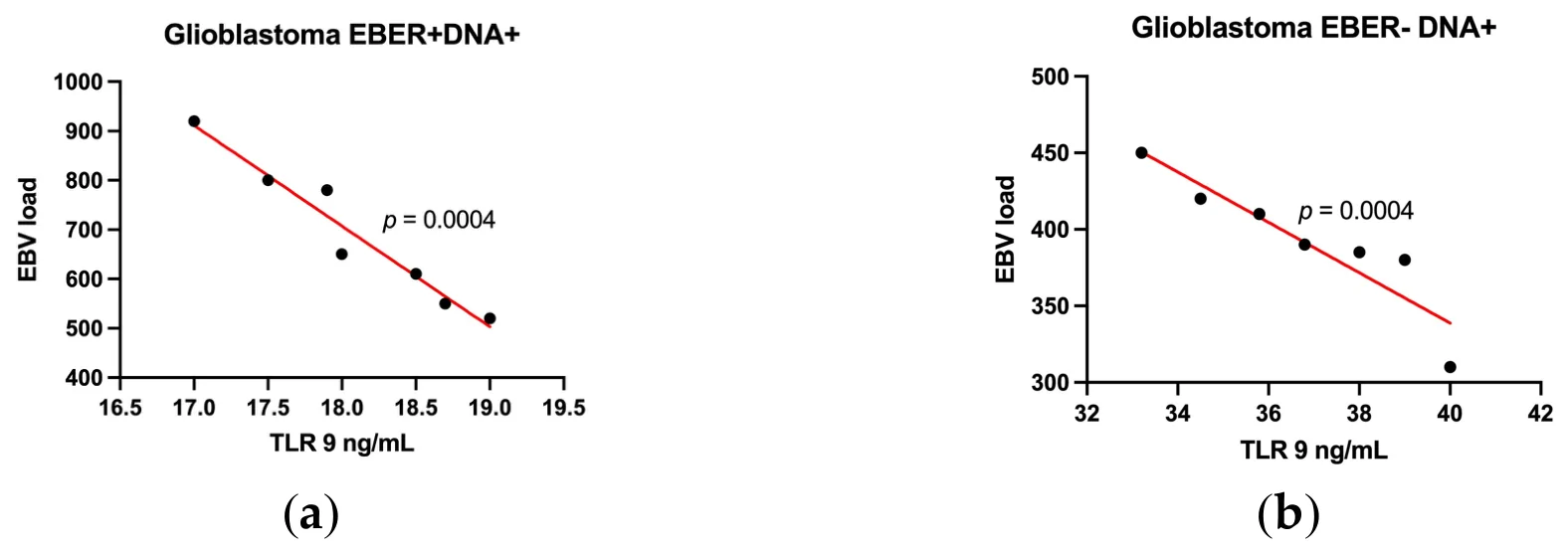
Analysis of correlation between TLR9 serum concentration and EBV load in glioblastoma: (**a**) EBER+DNA+; (**b**) EBER-DNA+.

In the next stage, we investigated a potential correlation between selected parameters, as well as the existence and nature of the correlation between TLR9 levels and EBV load in tumor tissue (Figure 6). In this instance, clinical samples from the EBER+DNA+ (Figure 6a) and EBER-DNA+ (Figure 6b) subgroups were examined.

As can be observed, higher viremia values corresponded to lower serum TLR9 levels in both the EBER-DNA+ and EBER-DNA+ groups.

**Figure 7:**
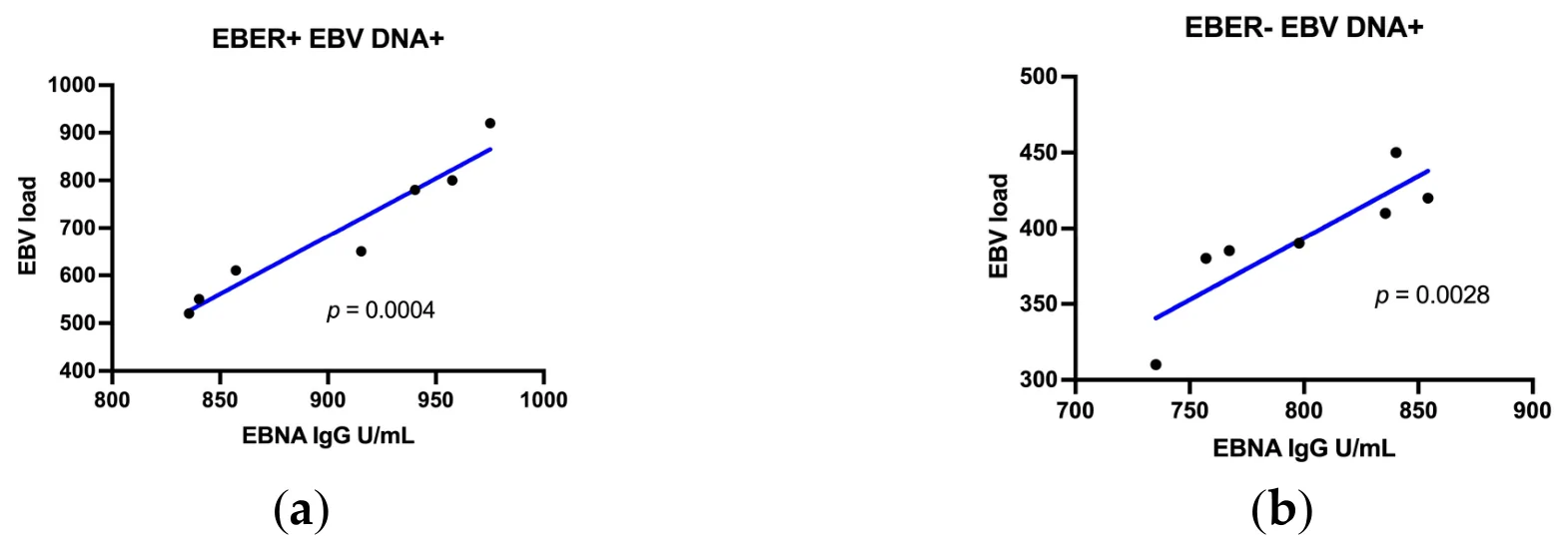
Correlations between EBV load and anti-EBNA antibody levels in glioblastoma patients: (**a**) EBER+ EBV DNA-; (**b**) EBER- EBV DNA+.

Correlations between EBV load and anti-EBNA antibody levels were evaluated in the same patient subgroups (Figure 7).

In this case, the levels of IgG antibodies against EBNA increased in parallel with viremia.

### 2.7. ROC (Receiver Operating Characteristic) Analysis: Evaluation of Diagnostic Accuracy of Serum TLR9 Concentration in Glioblastoma Patients, Stratified by the Presence of EBER and/or EBV DNA

The final part of our study involved evaluating the diagnostic utility of detecting serum TLR9 concentration in glioblastoma patients in the presence of EBER and/or EBV DNA by using ROC curve analysis. We considered both cases where EBER was detected in tumor tissue and cases where EBER was absent but EBV DNA was detected.

**Figure 8:**
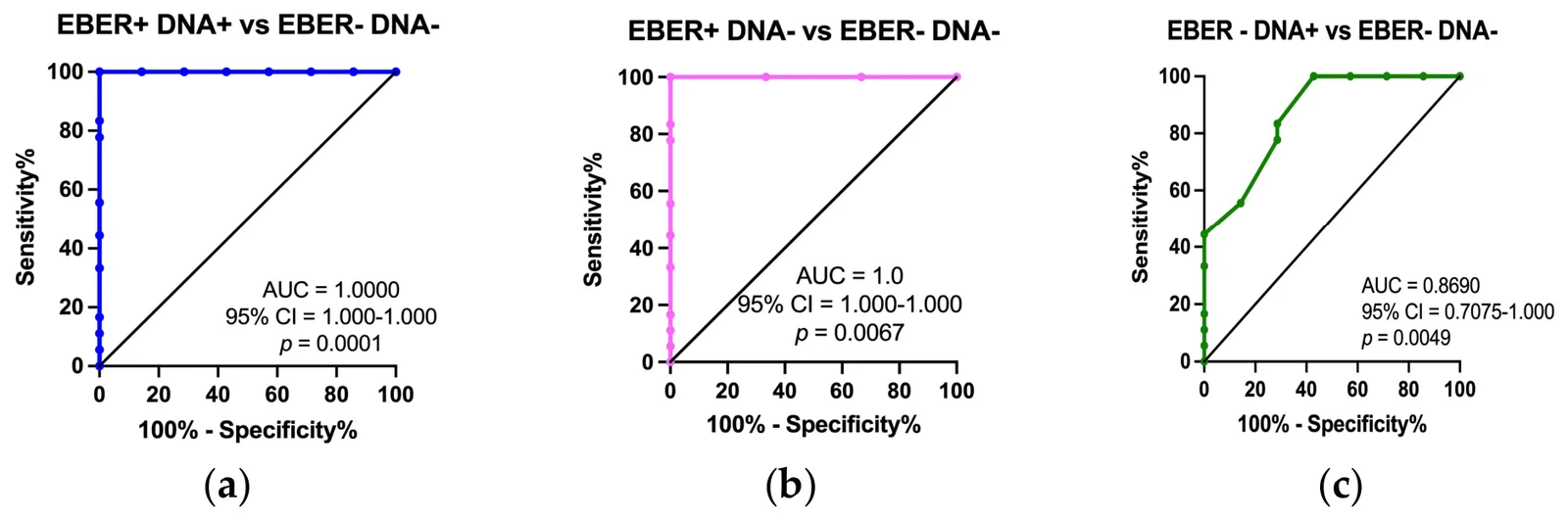
Evaluation of the diagnostic accuracy of TLR9 detection in glioblastoma patients by EBV status: (**a**) EBER+DNA+ vs. EBER-DNA-; (**b**) EBER+DNA- vs. EBER-DNA-; (**c**) EBER-DNA+ vs. EBER-DNA-.

The EBER+/EBV DNA+ (Figure 8a), EBER+/EBV DNA- (Figure 8b), and EBER-/EBV DNA+ (Figure 8c) subgroups were compared with the EBER-/EBV DNA- subgroup.

In the first case (Figure 8a), the ROC curve analysis revealed significant differences in TLR9 concentration, enabling us to effectively differentiate between EBV-positive and EBV-negative samples; the AUC value was 1.0, indicating excellent discriminative ability (*p* = 0.0001). In the second case (Figure 8b), the AUC value was also 1.0, indicating similar excellent discriminative ability (*p* = 0.0067). In contrast, the AUC for TLR9 detection when comparing EBER-/EBV DNA+ vs. EBER-/EBV DNA- was 0.8690 (*p* = 0.0049), indicating slightly lower diagnostic power (Figure 8c). The results of this analysis are summarized in Table S7 in the Supplementary Materials. As the analysis shows, TLR9 could be a marker for differentiating EBV-positive and EBV-negative glioblastoma patients.

## 3. Discussion

Numerous scientific studies have investigated the expression of TLR2 and TLR9 on glioma cells, highlighting the role of these receptors in promoting tumor progression [14,15,16,25,26,27,28].

In glioblastoma, the expression of both TLRs has been linked with tumor cell proliferation, invasion, angiogenesis, and the immunosuppressive remodeling of the tumor microenvironment [29,30,31,32,33,34,35].

**Figure 9:**
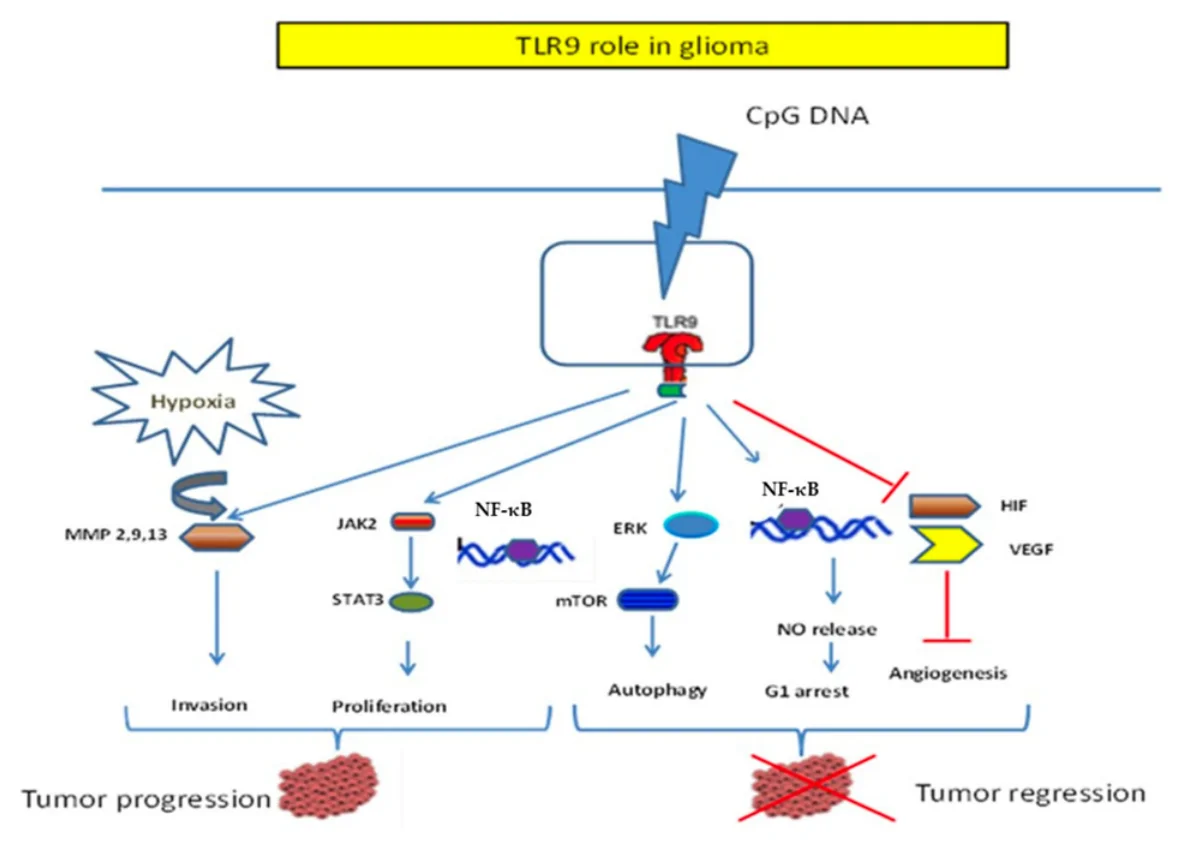
The dual role of TLR9 in glioma [40].

In the central nervous system, TLR9 is expressed in neurons, glial cells, and immune cells [36], playing a significant role in tumor cell function and the regulation of the immune response in glioma, which makes it a potential therapeutic target in cancer [20,37,38]. On the one hand, it can inhibit tumor growth, while on the other, it may promote tumor progression [39], a dual role that has been described in detail by Fehri et al. [40] and is illustrated in Figure 9.

The current study is a continuation of our earlier research but considered a slightly different group of patients [41]. We evaluated our results in the context of possible EBV infection.

Among known oncogenic viruses, human herpesviruses (HHVs) can exhibit neurotropism—manifested by the ability to cross the blood–brain barrier—and may contribute to the development of neurodegenerative diseases [42,43]. The potential association between EBV and glioma remains controversial, with some authors considering it a possibility and others rejecting it [44,45].

To investigate this association, the analyzed cohort was further stratified into four subgroups by EBV DNA status based on the EBER test results provided by a hospital pathologist.

Despite numerous experimental studies on TLR signaling pathways in glioblastoma, relatively little is known about their levels in the systemic circulation or their association with markers of the antiviral immune response. Determining whether the serum concentrations of these receptors reflect tumor-associated innate immune activity could provide clinically relevant, minimally invasive biomarkers of disease biology.

Therefore, in this study, we determined serum TLR2 and TLR9 concentrations in patients with this tumor and found higher levels in patients with brain cancer compared with the control group. Further, serum TLR2 concentrations in patients with glioblastoma and astrocytoma did not differ significantly, whereas the serum TLR9 concentration in glioblastoma patients was lower than in astrocytoma patients. In this respect, our results are consistent with the observations by the aforementioned authors.

The analysis showed that the presence of EBV did not affect TLR2 levels, which were similar across all analyzed subgroups, with slight differences that were not statistically significant. Moreover, all astrocytoma cases tested negative for EBER. It is possible that EBV-related signals originate from inflammatory cells or adjacent lymphoid cells.

However, significant differences in TLR9 levels were observed between the various subgroups. The lowest TLR9 concentration was recorded in the serum of patients whose tumor tissue tested positive for EBER, regardless of the presence or absence of EBV DNA. Numerous studies indicate that TLR9 overexpression is associated with a higher grade of glioma malignancy and a poorer prognosis [15,16,21,46].

Therefore, in the subsequent analysis, we focused exclusively on assessing TLR9 levels in patients with glioblastoma. We also evaluated EBV viremia in tumor tissue from glioma patients, who were divided into two subgroups: EBER+ and EBER-. Significantly higher EBV levels were observed in the EBER-positive samples.

It should be emphasized that in the present study, serum TLR9 was evaluated as a circulating biomarker rather than as a direct indicator of TLR9 expression in tumor tissue. Therefore, differences in serum TLR9 concentrations should not be interpreted as direct evidence of altered intra-tumoral TLR9 expression or the activation of the TLR9 signaling pathway. Nevertheless, the observed association between circulating TLR9 levels and EBV-related parameters may indicate a link between the innate immune response and EBV infection status in patients with glioblastoma. Further studies directly assessing TLR9 expression in tissues and downstream signaling components are required to elucidate the mechanisms underlying this phenomenon.

The evasion of host immune defense mechanisms and chronic infection are hallmarks of EBV-associated diseases. Many authors have demonstrated that the host immune response to viral infection plays a significant role in the process of tumorigenesis and, consequently, in tumor progression [47,48].

TLR9 is a key factor involved in the interaction between EBV and the host immune system and may influence neoplastic transformation [49,50]. Latent EBV infection can significantly inhibit TLR9 expression, a process mediated by the major EBV oncoprotein, LMP 1 [51,52,53,54]. Conversely, studies conducted by Liu et al. [55] demonstrated that TLR9 expression was reduced in B lymphocytes but elevated in monocytes in children with infectious mononucleosis (IM). These findings suggest that the impact of EBV on TLR expression may depend on cell-specific characteristics and individual host factors. Zhang et al. [56] described a novel mechanism by which EBV escapes immune system control: EBV can reduce TLR9 expression by disrupting RNA m6A modification. Moreover, EBNA1 affects METTL3, leading to altered TLR9 stability/expression, another mechanism which the authors demonstrated to be linked with immune evasion. Whether such mechanisms can explain the lower TLR9 concentration in our samples from patients with EBV requires further study.

The interaction between EBV and the human immune system is complex, as the virus possesses numerous mechanisms that allow it to evade control by both the innate and adaptive immune responses. This enables it to establish persistent latent infection in host cells; this can, however, transition into a lytic infection phase [57,58]. A key strategy the virus uses to evade the host’s immune response, known as “host shutoff” [59], can occur in two ways:

1. Canonical mechanism (cytoplasmic mRNA degradation). First, the BGLF5 nuclease cleaves cellular mRNA molecules in the cytoplasm; then, cellular exonucleases rapidly degrade the remaining mRNA fragments.

2. Non-canonical mechanism (loss of chromatin accessibility). EBV immediate-early protein Zta (also known as ZEBRA or BZLF1) initiates the transition from the latent to the lytic phase and limits host chromatin accessibility, inhibiting the transcription of cellular genes.

Assessing EBV DNA levels in conjunction with EBV-specific antibodies provides complementary information regarding the relationship between the presence of the virus and the host’s immune response. While the detection of EBV DNA indicates the presence of viral genetic material in tumor tissue, anti-EBVCA and anti-EBNA antibodies provide information on the body’s humoral response to EBV infection.

We observed that in the subgroup of patients testing negative for EBER-DNA, anti-EBNA and anti-EBVCA antibody levels were similar to those found in the control group and lower than in the other GB subgroups. The highest anti-EBNA antibody levels were observed in the EBER+ DNA+ subgroup, followed by the EBER+ DNA− and EBER− DNA+ subgroups.

Consequently, the simultaneous assessment of these parameters may provide a more comprehensive picture of the interaction between EBV and the host organism in the course of glioblastoma. Elevated EBV DNA levels in tumor tissue, accompanied by high concentrations of anti-EBVCA and/or anti-EBNA antibodies, may suggest that the presence of the virus within the tumor is associated with an established systemic antiviral response. However, it must be emphasized that EBV DNA detection and the strength of the humoral response are distinct biological processes and should not be regarded as direct equivalents. Persistent or latent EBV infection can be associated with the presence of detectable viral DNA, even with an established humoral immune response. From the perspective of the biological mechanism, the relationship between EBV DNA present in tissues and circulating EBV-specific antibodies can also be considered in the context of pathogen recognition by the innate immune system. TLR9 recognizes unmethylated DNA rich in CpG sequences and may play a role in the detecting viral DNA via innate immune mechanisms. Consequently, alterations in TLR9 expression or circulating levels of the receptor could potentially influence the host response to EBV infection and the persistence of viral genetic material within the tumor microenvironment.

The co-occurrence of detectable EBV DNA, EBV-specific antibodies, and altered TLR9 levels may therefore reflect complex interactions of viral persistence, innate immune recognition, and the adaptive humoral response in the context of glioma. Our results demonstrate an association between EBV-associated markers detected in tumor tissue and circulating TLR9 concentrations, but they do not establish that the EBV infection of glioma cells directly regulates TLR9 levels.

In the final stage of this study, we investigated potential correlations between selected parameters, as well as the existence and nature of the relationship between TLR9 levels and EBV viral load in tumor tissue. The results indicate that TLR9 may serve as a marker for differentiating glioblastoma patients based on their EBV infection status (positive or negative), as it was observed that in both EBER-DNA+ groups, higher viral load values were accompanied by lower serum TLR9 concentrations. Correlations between EBV viral load and anti-EBNA antibody levels were also assessed in these patient subgroups, and it was found that the IgG anti-EBNA antibody levels increased in parallel with the viral load.

ROC analysis made it possible to determine the diagnostic accuracy of serum TLR9 measurements in glioblastoma patients, taking into account both cases where EBER was detected in the tumor tissue and those where EBER was absent but EBV DNA was detected. However, these seemingly perfect estimates should be interpreted with great caution, and further research is needed.

EBER, detected with in situ hybridization (ISH) in EBV-associated tumors, revealed the presence and localization of small RNA molecules encoded by the virus within the tissue. However, as indicated by data from the literature, reduced EBER expression may occur in EBER−/EBV DNA+ patients, especially in advance stages of cancer [60]. Li et al. [60] identified the potential prognostic value of the combination of EBER and EBV DNA in NPC, as they observed a discrepancy between EBER status and the detectability of EBV DNA in some patients with nasopharyngeal carcinoma (NPC). Given the established role of EBV infection in the pathogenesis of NPC, these findings provide a basis for refining clinical management in patients with EBV-negative nasopharyngeal carcinoma. Nevertheless, whether our results indicate a similar relationship requires confirmation in further studies.

EBV DNA detected via PCR may originate from infiltrating EBV-positive lymphocytes or other non-neoplastic cellular elements, whereas the absence of detectable EBER molecules may indicate low levels of EBV transcription, variable viral activity, or the presence of viral DNA in cells lacking EBER expression. Consequently, in the present study, the results regarding EBER and EBV DNA can be interpreted as complementary indicators of the presence of EBV-associated markers in the tumor tissue, rather than as definitive evidence of direct EBV infection of the glioma cells.

These considerations must take into account the significant role of the microbiome, which can either promote or inhibit tumor development and progression by interacting with cancer cells or the host’s immune system, thereby influencing the efficacy of anti-cancer therapy [61,62]. In addition, the significant role of genetic and epigenetic alterations in the pathogenesis of glioblastoma—influencing cell proliferation, differentiation, and invasiveness, as well as the response to treatment—must also be emphasized [63]. When interpreting these results, the molecular characteristics of the tumors must also be considered. In our cohort, glioblastomas were IDH-wildtype, whereas astrocytomas were IDH-mutant, reflecting two biologically distinct molecular entities. IDH status is associated with significant differences in tumor biology, epigenetic regulation, tumor evolution, and the composition of the tumor microenvironment. As noted by Tralongo et al. [63] in their recent review, molecular heterogeneity can persist within individual IDH-defined entities, including differences in DNA methylation patterns and other genomic and epigenetic alterations. Therefore, the differences in TLR2 and TLR9 levels observed between glioblastoma and astrocytoma should be interpreted in the context of both histological classification and molecular status. In contrast, the observed association between EBV positivity and lower levels of circulating TLR9 in the glioblastoma group represents a distinct finding and may reflect interactions between EBV and innate immunity within the specific molecular and immunological context of IDH-wildtype glioblastoma. However, since this study was not designed to investigate the impact of molecular changes on the relationship between EBV and TLR9, this interpretation remains a hypothesis requiring further verification.

Should the role of EBV in glioblastoma really be ruled out? Akhatar et al. [64] raise the question of whether low EBV load within these tumors drives oncogenesis.

**Figure 10:**
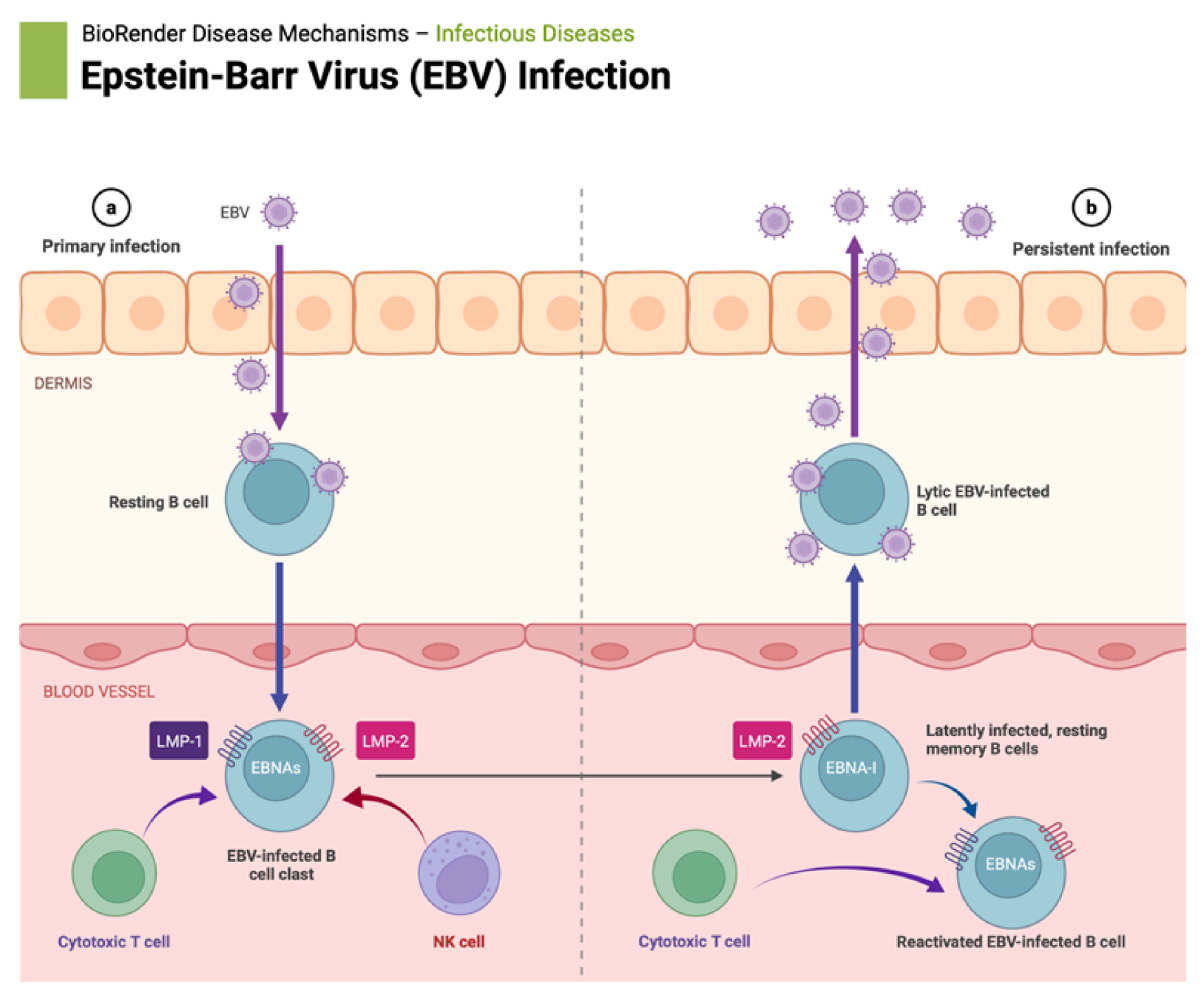
Epstein–Barr virus infection. Created in BioRender. Polz-Dacewicz, M. (2026) (https://BioRender.com/csj4tdi).

Lytic replication is a significant risk factor for the development of EBV-associated malignancies [65]. The primary and persistent EBV infection processes are represented in Figure 10.

Shumilov et al. [66] reported that EBV may exert oncogenic effects without establishing latency (e.g., by inducing centrosome amplification and chromosomal instability) and may constitute a factor for glioma development, even when the virus is not detected in tumor tissue.

There are also studies suggesting that EBV reactivation may occur as a result of immunosuppression and/or stress associated with GB therapy. Zakaria et al. [67] described a patient who developed EBV-positive primary CNS lymphoma two months after undergoing radiochemotherapy for glioblastoma.

In the context of reduced TLR9 levels, this result may indicate impaired recognition by the innate immune system, despite an intensified systemic humoral response. Thus, there remains the question of whether impaired innate immune surveillance—conditioned by the altered immune microenvironment of the tumor—plays a potential role in the progression of glioblastoma.

**Figure 11:**
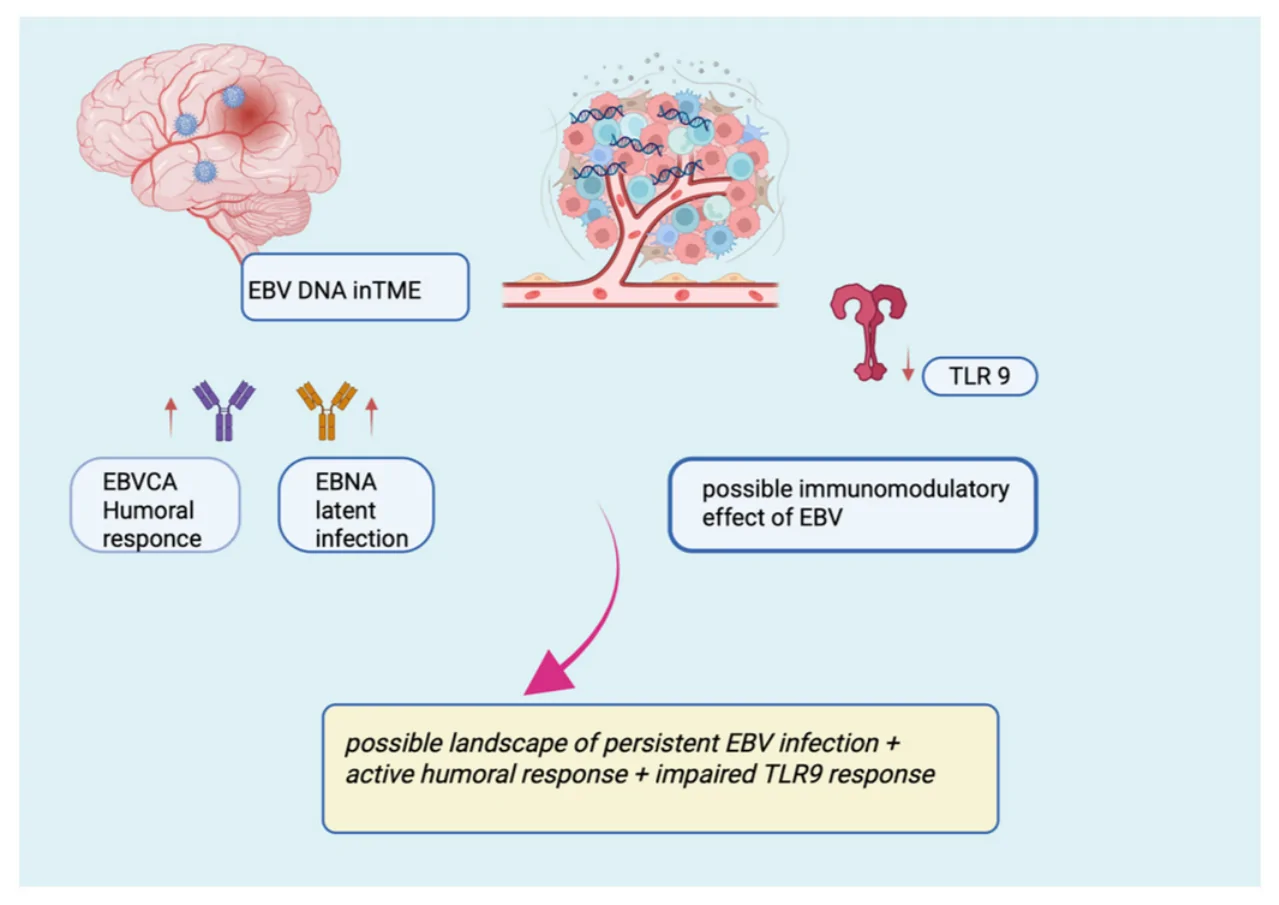
Possible mechanistic model of persistent EBV infection, active humoral response, and impaired TLR9 response. Created in BioRender. Polz-Dacewicz, M. (2026) (https://BioRender.com/oi5s8tu).

It can be hypothesized that in the case of glioblastoma (GB), EBV may induce a chronic humoral response—characterized by elevated levels of antibodies against EBV-VCA and EBNA antigens—while simultaneously modulating the innate response by inhibiting TLR9 activity or its signaling pathway. This mechanism may potentially promote EBV persistence within the tumor microenvironment and facilitate the evasion of immune surveillance. Since TLR9 was measured in serum and EBV DNA was measured in GB tissue, it cannot be directly concluded that EBV DNA in the tumor causes a decrease in TLR9; however, a biological association—which requires confirmation—can be posited. It should be emphasized that EBVCA IgG and EBNA IgG do not in themselves indicate active EBV replication—these antibodies can persist after a past infection. Elevated levels of these antibodies, in the context of high EBV DNA, may indicate stronger exposure to EBV or reactivation. Based on the obtained results, we propose a mechanistic model of the observed relationships among the studied parameters (Figure 11).

All these uncertainties point to the need for further, more detailed, and comprehensive research to clarify whether EBV plays a role in gliomagenesis and oncomodulation. Understanding the role of EBV in the etiology of gliomas could open up new possibilities for treating this cancer.

However, these results should be interpreted with caution, as the observed associations do not prove a causal link among EBV persistence, TLR9-mediated signaling, and the development or progression of glioma. Further research is required to elucidate the underlying mechanisms, including EBV transcriptional activity, viral localization within tumor cells, TLR9 expression in the tumor microenvironment, and longitudinal changes in the EBV-specific immune response.

### Limitations

Our research study presents some limitations. First of all, it should be emphasized that the presented study results reflect only a specific aspect of glioblastoma immunopathogenesis. It should also be noted that this study involved a limited number of patients, a constraint dictated by the availability of clinical material. In particular, the small size of certain subgroups may limit the precise assessment of the relationship between TLR9 levels and the other parameters analyzed. Consequently, the findings apply only to this specific group of patients and cannot be generalized.

This study should be considered preliminary. It did not monitor patients with glioblastoma (GB) in the long term, which precludes an assessment of changes in TLR9 levels over time and their potential impact on disease progression, treatment response, or the prognosis. Although the findings reflect the patients’ status only at the time of diagnosis, they provide a promising starting point for further, in-depth analysis. These limitations highlight the need for subsequent, more detailed studies involving larger patient cohorts to better understand the role of TLR9 in the pathogenesis of glioblastoma and its implications for treatment.

## 4. Materials and Methods

This study included a total of 49 patients with a diagnosed and histopathologically confirmed brain tumor, hospitalized at the Department of Neurosurgery, 1st Clinical Military Hospital with Outpatient Clinic, in Lublin, Poland.

The study group consisted of 35 patients (71.4%) with *IDH-wildtype glioblastoma* (grade 4) and 12 patients (28.6%) with *IDH-mutant* *astrocytoma* (grade 2). Forty sex- and age-matched healthy volunteers served as the control group. Table 1 presents the basic characteristics of both groups.

Tumor tissue samples were collected during a neurosurgical operation. All clinical samples were examined by an experienced hospital pathologist in accordance with hospital procedures as follows: histopathological assessment (grading) was performed in accordance with the WHO classification of tumors of the central nervous system (CNS) published in 2021 [68,69]; in situ hybridization (ISH) was performed to analyze RNA encoded by the Epstein–Barr virus (EBER)—the gold standard for detecting and localizing latent EBV in biopsies and cancer tissues.

### 4.1. Clinical Samples

The clinical material collected from the patients with brain cancer included blood and tumor tissues. Following histopathological assessment and EBER analysis by an experienced hospital pathologist, we received clinical samples of tumor tissue, which we analyzed in our laboratory for the presence of EBV DNA.

#### DNA Extraction and Detection

DNA isolation was performed using the QIAamp DNA Mini Kit (Qiagen, Hilden, Germany), according to the manufacturer’s instructions. The quality of the extracted DNA was then assessed by performing a β-globin test, which was used both to ensure DNA integrity and to identify the presence of potential PCR inhibitors.

For DNA amplification, the GeneProof Epstein–Barr virus (EBV) PCR Kit; IVD (GeneProof, Brno, Czech Republic) was used in accordance with the manufacturer’s protocol (real-time qPCR method). All samples were tested twice, and EBV load was expressed as copies per 1 µg of tissue DNA. The detailed procedure was described in our earlier publication [41].

### 4.2. Serum Sample Collection

The concentrations of TLRs and anti-EBV antibodies in the serum of the patients and control subjects were evaluated. Venous blood collected from all subjects was centrifuged at 1500 rpm for 15 min at room temperature, aliquoted, and frozen (−80 °C) until analysis.

### 4.3. Serological Methods—Detection of TLRs and Anti-EBV Antibodies

The serum concentrations of TLR2 and TLR9 were quantified using commercially available ELISA kits by Cloud-Clone Corp (Houston, TX, USA; SEA663Hu and SEA709Hu), which can be used to quantitatively determine TLR2 and TLR9 in human serum, plasma, tissue homogenates, cell lysates, cell culture supernatants, and other body fluids. The TLR concentrations in the samples were determined by comparing their optical density (O.D.) with a standard curve.

Absorbance was measured using a Labexim Ledetect 96 microplate reader (Lengau, Austria), and the results, presented in ng/mL, were analyzed using MicroWin 2013 (Lite+) software. The minimum detectable concentrations were 0.112 ng/mL for TLR2 and 0.056 ng/mL for TLR9.

Anti-EBV antibodies were detected using the commercially available Microblot-Array EBV IgG test kit (TestLine Clinical Diagnostics s.r.o., Brno, Czech Republic—IVD CE), as previously described [41].

### 4.4. Statistical Analysis of Results

All statistical analyses were performed using GraphPad Prism 10.4.0 (GraphPad Software, San Diego, CA, USA) and Tibco Statistica 13.3 (StatSoft, Kraków, Poland). The Shapiro–Wilk test was applied to evaluate the normality of the continuous data. Categorical variables were compared using the Pearson chi-squared test or, where appropriate, Fisher’s exact test. Continuous variables with non-normal distributions were compared using the Mann–Whitney U and Kruskal–Wallis tests, depending on the number of groups.

Correlations between the variables were determined using Spearman’s rank correlation coefficient. A *p*-value ≤ 0.05 was considered statistically significant.

## 5. Conclusions

Numerous scientific reports indicate that Toll-like receptors (TLRs) play a significant role in the tumor microenvironment, including in brain tumors, as their activation can both support an anti-tumor response and promote tumor progression through chronic inflammation. Despite significant progress in understanding the functions of these receptors, the precise molecular mechanisms of their action remain unclear, a fact stemming from the complexity and interconnectedness of their roles within innate and adaptive immunity.

Our results show that TLR2 and TLR9 levels were higher in patients with glioblastoma compared with the control group. Interestingly, however, TLR9 concentrations were lower in EBV-positive patients. Furthermore, a detailed analysis revealed an association between TLR9 levels and the presence of EBER and/or EBV DNA in tumor tissue.

Although these findings indicate a potential link between the TLR9-dependent immune response and EBV infection in glioblastoma, a direct causal relationship between EBV and TLR9 regulation cannot be established and requires further confirmation. Considering the still-unclear role of EBV in glioblastoma pathogenesis, further studies are needed to elucidate the underlying mechanisms and determine the biological significance of this association.

## Figures and Tables

**Table 1:** Basic characteristics of the study and control groups.

		N = 49	%	N = 40	%	*p*-Value
Sex	Male	29	59.2	25	62.5	0.8288
	Female	20	40.8	15	37.5	
Age	50–59	23	46.9	19	47.5	0.9999
	60–79	26	53.1	21	52.5	
Place of residence	Urban	22	44.9	19	47.5	0.8336
	Rural	27	55.1	21	52.5	
Smoking	Yes	13	26.5	14	35.0	0.4879
	No	36	73.5	26	65.0	
Alcohol abuse	Yes	14	28.6	10	25.0	0.8118
	No	35	71.4	30	75.0	
Type	Glioblastoma Grade IV	35/49 (71.4%)				
	Astrocytoma Grade II	14/49 (28.6%)				

## Data Availability

The original contributions presented in this study are included in the article/Supplementary Materials. Further inquiries can be directed to the corresponding author.

## Supplementary Materials

The following supporting information can be downloaded at: https://www.mdpi.com/article/10.3390/ijms27188408/s1.

## Abbreviations

The following abbreviations are used in this manuscript:

CNS	central nervous system
DAMPs	damage-associated molecular patterns
DC	dendritic cell
EBER	Epstein–Barr virus-encoded small RNAs
EBV	Epstein–Barr virus
EBVCA	Epstein–Barr virus capsid antigen
EBNA	Epstein–Barr nuclear antigen
GB	glioblastoma
ISH	in situ hybridization
LMP1	Latent Membrane Protein 1
MyD88	Myeloid differentiation factor 88
NF-κB	Nuclear factor kappa B
NPC	nasopharyngeal carcinoma
PRRs	pattern-recognition receptors
TLR	Toll-like receptor
TME	tumor microenvironment
