# Supplementary material for: TLR2 and TLR9 in Brain Cancer Patients in Relation to EBV Status
Source: Int J Mol Sci. 2026 Sep 21;27(18):8408. doi: 10.3390/ijms27188408 (PMC13607434; doi:10.3390/ijms27188408)
Supplement: Supplementary file 1 [file ijms-27-08408-s001.zip › ijms-4578577-supplementary.pdf]

Supplementary Materials

Table S1. Serum level of TLR 2 and TLR 9 in patients with glioblastoma, astrocytoma compared to control group (ng/mL).

TLR 9	Mean	Me	Min-Max	SD	*p* value
Glioblastoma	33.5	38.0	17.0 – 48.0	10.2	
Astrocytoma	80.1	82.0	65.0 – 90.0	8.2	<0.0001*
Controls	4.3	4.1	3.5 – 5.5	0.7	
p					
TLR 2					
Glioblastoma	65.5	67.0	49.0 – 70.0	65.5	
Astrocytoma	62.9	67.0	49.0 – 70.0	62.9	<0.0001*
Controls	5.9	5.8	4.9 – 6.9	5.9	
p	GB vs Astrocytoma	0.6368			

*Statistically significant

Table S2. Serum level of TLR 9 in GB patients according to EBER and DNA (ng/mL)

Parameter	EBER+ DNA+	EBER+ DNA-	EBER- DNA+	EBER- DNA-	*p*
Mean	18.1	18.2	36.7	40.7	
Median	18.0	18.7	36.8	40.0	0.0001*
Min-Max	17.0 – 19.0	17.0 – 19.0	33.2 – 40.0	38.0 – 48.0	
SD	0.7	1.1	2.4	2.7	
p	0.7333		0.0030*		

*Statistically significant

Table S2a. Serum level of TLR 9 in Astrocytoma patients according to EBER and DNA (ng/mL)

Parameter	EBER-DNA+	EBER-DNA-	*p* value
Mean	78.8	80.8	
Median	79.0	84.0	
Min-Max	65.0 – 90.0	67.0 – 89.0	0.5035
SD	8.9	8.3	

Table S3. Serum level of TLR 2 in GB patients according to EBER and DNA (ng/mL)

Parameter	EBER+ DNA+	EBER+ DNA-	EBER- DNA+	EBER- DNA-	*p*
Mean	64.7	67.3	65.8	65.4	
Median	67.0	68.0	65.0	66.0	0.8236
Min-Max	49.0 – 70.0	65.0-69.0	62.0 – 70.0	49.0 – 70.0	
SD	7.3	2.1	2.8	4.7	
p		0.8500		0.8701	

*Statistically significant

Table S3a. Serum level of TLR 2 in Astrocytoma patients according to EBER and DNA (ng/mL)

Parameter	EBER-DNA+	EBER-DNA-	*p* value
Mean	78.8	80.7	
Median	79.0	84.0	
Min-Max	65.0 – 90.0	67.0 – 89.0	0.1818
SD	8.9	8.3	

Table S4. EBV load in the Studied Subgroups of Glioblastoma Patients (EBER+ and EBER-) and comparison glioblastoma and astrocytoma in EBER-DNA+ samples.

Studied group	Mean	Min-max	SD	P value
EBER-DNA+ Glioblastoma	392.1	310.0 – 450.0	43.5	0.0025*
EBER-DNA+ Astrocytoma	208.0	150.0 – 280.0	56.3	
GB EBER+DNA+	650.0	520.0 – 920.0	147.0	0.0006*
GB EBER-DNA+	392.0	310.0 – 450.0	43.6	

*Statistically significant

Table S5. Serum EBNA IgG level in glioblastoma patient subgroups (U/mL)

Parameter	EBER+ DNA+	EBER+ DNA-	EBER- DNA+	EBER- DNA-	Controls	*p*
Mean	903.1	803.7	798.2	386.8	335.3	
Median	915.4	800.2	797.8	384.9	306.1	0.0001*
Min-Max	835.6 – 975.2	790.3 – 820.5	735.2 – 854.2	349.8 – 475.4	280.5 – 495.5	
SD	58.2	15.4	46.4	32.4	61.8	
p		0.0167*		0.0001*	0.0008	

*Statistically significant

Table S6. Serum EBVCA IgG level in glioblastoma patient subgroups (U/mL)

Parameter	EBER+ DNA+	EBER+ DNA-	EBER- DNA+	EBER- DNA-	Controls	*p*
Mean	969.2	775.1	888.1	378.8	375.5	
Median	969.4	775.3	870.8	380.3	380.3	0.0001*
Min-Max	942.9 – 992.5	751.6 – 798.3	851.6 – 985.4	280.5 – 585.4	280.5 – 585.4	
SD	19.0	23.4	46.6	91.2	88.3	
p		0.0167*		0.0001*	0.9234	

*Statistically significant

Table S7. Tabulated summary of ROC curves for TLR 9 in GB patients

	EBER+ DNA+	EBER+ DNA-	EBER- DNA+
Area	1.000	1.000	0.8690
Std. Error	0.000	0.000	0.08242
95% CI	1.000-1.000	1.000-1.000	0.7075-1.000
p value	0.0001	0.0067	0.0049
